# Protein crystallization screens developed at the MRC Laboratory of Molecular Biology

**DOI:** 10.1016/j.drudis.2016.03.008

**Published:** 2016-05

**Authors:** Fabrice Gorrec

**Affiliations:** MRC Laboratory of Molecular Biology, Francis Crick Avenue, Cambridge Biomedical Campus, Cambridge CB2 0QH, UK

## Abstract

In order to solve increasingly challenging protein structures with crystallography, crystallization reagents and screen formulations are regularly investigated. Here, we briefly describe 96-condition screens developed at the MRC Laboratory of Molecular Biology: the LMB sparse matrix screen, Pi incomplete factorial screens, the MORPHEUS grid screens and the ANGSTROM optimization screen. In this short review, we also discuss the difficulties and advantages associated with the development of protein crystallization screens.

## Highlights

• We present three types of initial screens and an optimization screen for protein crystallization.
• Published conditions were selected to formulate the LMB sparse matrix.
• Pi screens are composed of maximally diverse sets of conditions.
• MORPHEUS screens integrate mixes of additives selected from the Protein Data Bank.
• ANGSTROM was formulated with polyols for later optimization of crystals.

## Introduction

X-ray crystallography is extensively applied to solve the structures of biological macromolecules, notably proteins, their complexes and assemblies. Recent developments in new X-ray sources and beamlines have enabled different approaches to data collection [1]. In addition, other techniques that can be used to solve structures utilizing crystals are also being developed, for example electron microscopy [2]. The resulting structures are essential to our understanding of biological mechanisms at the atomic level and assist rational drug design [3]. Nevertheless, protein crystallization experiments usually generate low yields of crystals with sufficient quality to solve structures. An underlying reason for the low yield of such crystals is the large number of combinations of variables associated with successful protein crystallization [4]. In addition to problems relating to the stability, shapes and surfaces of the proteins, one has to consider experimental parameters such as pH, temperature, physicochemical properties of the conditions, among others. Further problems arise subsequently with the cryo-cooling of crystals (required to reduce radiation damage during data collection) and in data processing (since crystals are often not sufficiently well-ordered).

Typically, crystallization involves a solution that includes three types of reagents: a precipitant, a buffer-controlling pH and an additive. A condition can be seen as a combination that alters the multitude of variables associated with crystallization experiments. There are now hundreds of well-known crystallization reagents and, hence, a systematic permutation of these reagents (as in factorial or grid screens), at various concentrations, would include millions of unique combinations. However, the implementation of such a comprehensive screening program is prevented by restricted sample quantity and the time associated with the crystallization setup. Two main formulation approaches of initial screens have been implemented to reduce the number of trials: incomplete factorial and sparse matrix. For the incomplete factorial approach, conditions are formulated *de novo* in accordance with the two principles of randomization and balance, as suggested originally by Carter and Carter for all the main parameters related to the crystallization of tryptophan tRNA synthetase [5]. The first widely used sparse matrix screen was developed by Jancarik and Kim and involved a selection of 50 conditions that were found to have been successful with homogeneous samples of various kinds of proteins [6]. Over the past decade, another approach has also been employed that consists of integrating mixes of additives into the formulation of an initial screen (the ‘silver bullets’ approach) [7]. After initial hits have been obtained, in most cases one has to attempt to reproduce the crystals and optimize their diffraction. To do this, the physicochemical properties of the sample, the initial conditions employed and the cryo-cooling of crystals could be fine-tuned with an additive screen [8].

The robotic nanoliter protein crystallization facility at the Medical Research Council Laboratory of Molecular Biology (MRC LMB, Cambridge, UK) supports more than 70 users [9]. A main feature of this facility is the availability of 96-condition crystallization plates pre-filled with a broad variety of screening kits for vapor-diffusion experiments. The entire set of our pre-filled plates can be used to form a large initial screen against a novel sample (20 plates since 2015, *i.e*. 1920 conditions), or just a few plates can be selected to match specific requirements [10]. This context is ideal to investigate crystallization reagents and screen formulations. Here, we briefly describe 96-condition screens developed in our facility: (i) the LMB sparse matrix [11]; (ii) Pi incomplete factorial screens [12]; (iii) the MORPHEUS grid screens integrating cryo-protected conditions made up of multicomponent mixes 13, 14; and (iv) the ANGSTROM optimization screen that is exclusively composed of polyols. In this short review, we also discuss the difficulties and advantages associated with the development of crystallization screens.

## LMB sparse matrix

Because of the unpredictable nature of protein crystallization, the development of screens has often been driven by empirical results. Since Jancarik and Kim's screen, the dramatic increase in availability of crystallization data has stimulated the optimization of sparse matrices biased toward DNA and RNA [15], transmembrane proteins [16] and other considerations such as cost-effectiveness [17]. Unavoidably, formulations of sparse matrices are also biased toward the subset of initial conditions and the approach to crystallization employed.

We studied the published conditions for crystal growth that resulted in protein structures at the LMB between 2002 and 2009 [11]. In total, more than four million individual crystallization experiments (∼2800 samples) were set up following standard procedures with the vapor-diffusion technique and an initial screen then composed of 15 pre-filled plates (*i.e.* 1440 conditions). The average molecular weight of the crystallized proteins was 37 kDa, including large complexes of 100–200 kDa. Published results with transmembrane proteins and samples containing long nucleic acids (RNA or DNA) were excluded in this study because they have very different physicochemical properties and hence generally require different approaches.

Although the original purpose of our study was a statistical analysis, a different application later emerged with the selection of 96 non-redundant conditions that formulated a sparse matrix for soluble proteins and their complexes with relatively high molecular weights. Table S1 (see supplementary material online) shows the formulation for the LMB sparse matrix screen and all the other screens presented later (the format is database-friendly).

**Figure 1:**
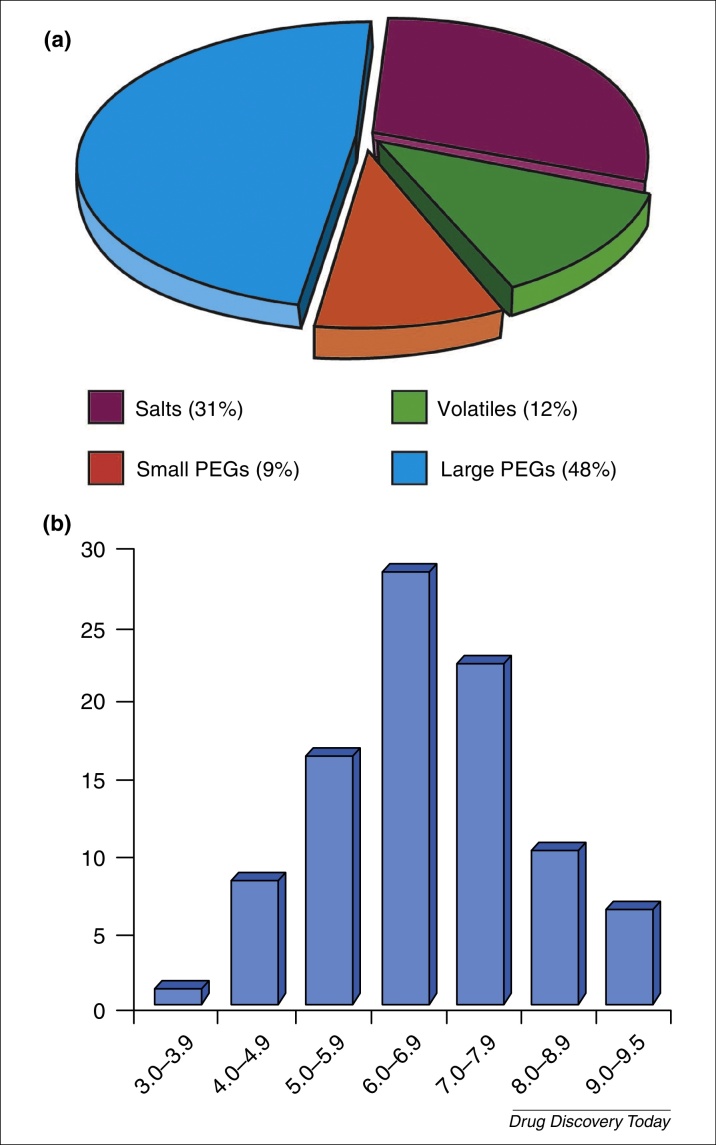
**(a)** Pie chart showing the crystallization propensities of the main groups of precipitants. The precipitants are found in 96 typical conditions (*i.e.* conditions with a single precipitant) that were optimized at the MRC Laboratory of Molecular Biology (LMB). Cryo-protectants (essentially glycerol) were excluded from the analysis. Large polyethylene glycols (PEGs) are highly successful. **(b)** Occurrences of pH value clusters. A wide range of pH values needs to be investigated to crystallize different types of samples (pH 3.0–9.5) with main occurrences of pH value clusters in the range 5.0–7.9. Note that five out of 96 conditions were not buffered (Table S1, see supplementary material online).

Polyethylene glycols (PEGs) were found to be the most successful precipitants (Fig. 1a), especially those with high molecular weight (MW ≥1000 Da; 46% of published conditions), followed by common salts (ammonium sulfate or phosphate, sodium citrate, others) and small volatiles (ethanol, 2-methyl-2,4-pentanediol, others). This trend has been observed elsewhere [18], although it might not apply to specific subsets of targets such as transmembrane proteins [19]. The optimum pH value clusters were in the range 5.0–7.9 (72% of published conditions, Fig. 1b), whereas the pH used to produce the samples is typically within the range 6.0–8.0. To some extent, this corresponds to an analysis produced elsewhere that emphasizes a well known correlation between crystallization pH and the isoelectric point of the protein [20].

## Pi incomplete factorial screens

**Figure 2:**
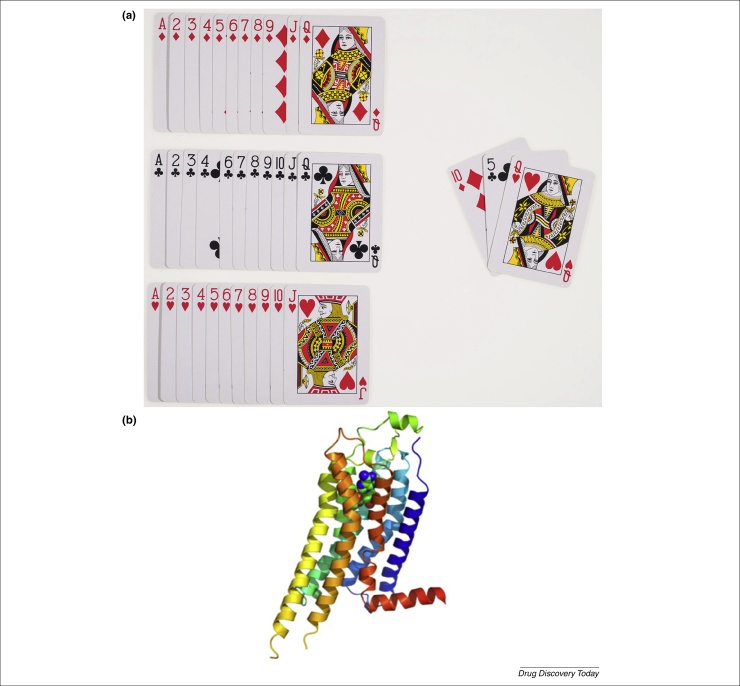
**(a)** Pi sampling represented with playing cards. Three sets of 12 cards (Kings excluded) represent the stock solutions grouped by class and sorted according to a main property. Unlike most card games, the aim is to generate maximally diverse sets of cards. The triplet on the right corresponds to the condition that will be found in well B10 according to the standard 96-condition plate layout. **(b)** Structure of the human adenosine A_2A_ receptor (A_2A_R-GL31) bound to its endogenous ligand adenosine. Diffraction-quality crystals of this thermostabilized G-protein-coupled receptor (GPCR) bound to different ligands were initially obtained with screens formulated following the Pi sampling strategy, notably the Pi-PEG screen [22]. Work of Guillaume Lebon (Institut de Génomique Fonctionelle, Montpellier, France).

In 2011, we published an incomplete factorial formulation method called Pi sampling that is specifically applied to the standard 96-condition plate layout (i.e. columns 1–12 and rows A–H) [12]. Pi sampling is intended to help laboratories on a day-to-day basis to formulate crystallization screens based on the properties of their macromolecules and the techniques employed for crystallization. Pi sampling uses modular arithmetic to generate maximally diverse combinations of three reagents. In Fig. 2a, the stock solutions are represented with playing cards and an example of a generated combination is shown. Each reagent comes from one of the three groups of 12 chosen stock solutions. Reagents are first grouped by class and sorted according to a main property (molecular weight, pH, hygroscopy, other). Diversity between the corresponding conditions is accentuated by varying the concentrations of reagents. Ninety-six combinations are then generated with a freely available web-based applet (http://pisampler.mrc-lmb.cam.ac.uk). The applet also generates the necessary information to prepare the corresponding Pi screen by hand or with an automated system. It should be noted that a program developed elsewhere can generate incomplete factorial screens with any chosen number of conditions [21].

A positive impact of Pi sampling on the crystallization of a G-protein-coupled receptor (GPCR) that had been difficult to crystallize previously (the adenosine A_2A_ receptor, construct A_2A_R-GL31; Fig. 2b) was observed when we formulated Pi screens such as Pi-PEG (Table S1, see supplementary material online) [22]. For the Pi-PEG, we took into consideration general observations made in previous works with GPCRs, which indicated that the use of screens formulated with PEGs and buffers gave a greater yield of crystals than all commercially available screens (at least for vapor-diffusion experiments). Recently, other laboratories have employed Pi sampling with success, notably during structural studies of proteins from Gram-positive pathogens 23, 24.

## MORPHEUS grid screens

**Figure 3:**
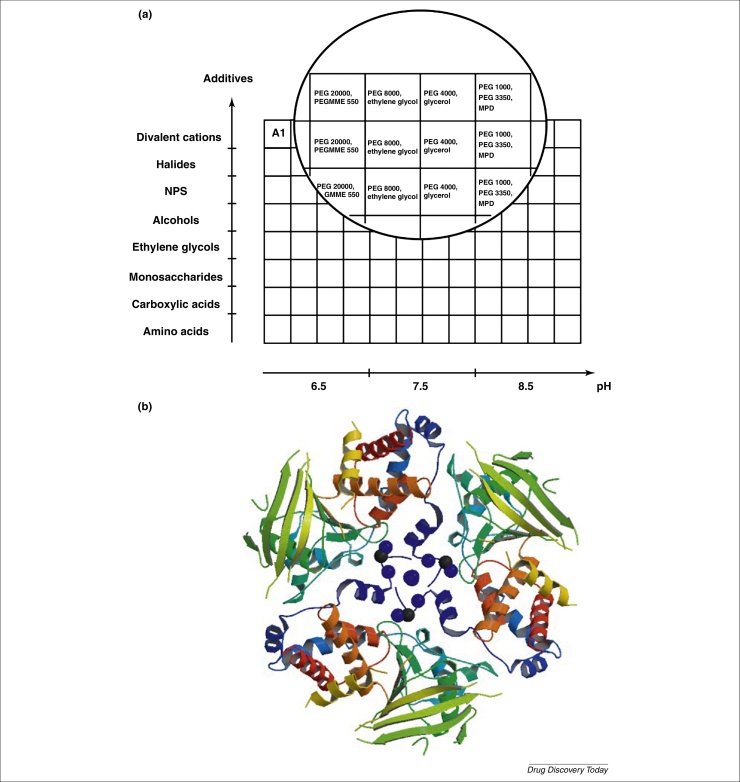
**(a)** MORPHEUS schematic screen layout. The layout shows a 3D grid screen with four precipitant mixes, eight additive mixes and three buffer systems (4 × 8 × 3 = 96 conditions) found in the original MORPHEUS screen. All three stock solutions, the ligand mixes, the precipitant mixes and the buffers are combined using a fixed volume ratio of 0.5 stock precipitants + 0.1 stock additives + 0.1 buffer-system + 0.3 water. The same approach to formulation was employed to formulate MORHEUS II with different reagents. **(b)** Structure showing a cross-linked human protein tyrosine phosphatase receptor type J (PTPNJ; PDB ID: 2CFV). The protein crystallized only as a trimer that is not observed naturally. The trimer is formed with interactions between divalent metal ions (blue spheres) and alpha-helix structures (His-tags, blue ribbons). Unpublished results obtained during the early stage of the MORPHEUS screen development [13]. With the permission of Alastair J. Barr (University of Westminster, London, UK).

The formulation of a MORPHEUS screen follows a 3D grid approach, where eight mixes of additives are combined with four precipitant mixes and three buffer systems. Fig. 3a shows the schematic screen layout formulation for the original MORPHEUS screen published in 2009 [13]. The use of mixes enables a more extensive screening of components with a positive contribution to crystallization [7]. Also, by selecting additives on their high occurrence in the Protein Data Bank as ligands (http://www.rcsb.org), the chances of incorporating one that stabilizes or cross-links the protein (Fig. 3b), or promotes crystallization in some other way, should be increased. Finally, more than one type of additive might be required for crystal growth, because many structures in the PDB exhibit proteins bound with multiple additives.

In 2015, a follow-up screen called MORPHEUS II was published [14]. MORPHEUS II integrates reagents not seen in other initial screens commercially available. Notably, heavy atoms were used (e.g. rare and alkali earth metals). Many heavy atoms are not very soluble and will usually destabilize a protein. Nevertheless, heavy atoms can opportunistically enable a crystal structure to be solved when they become part of the crystals initially produced without them through isomorphous replacement or even Single or Multi-wavelength Anomalous Diffraction (SAD or MAD) method [25].

In addition to additive mixes, the MORPHEUS screens integrate mixes of precipitants and buffer systems. Precipitants can be mixed to have a synergistic effect and to provide cryo-protection [26]. An advantage of buffer systems is that no concentrated acid or base is required to alter the pH and hence the formulation becomes fully amenable to automation. Initially, the original MORPHEUS screen [13] generated diffraction-quality crystals for projects such as phosphoinositide 3-kinase [27] and ubiquitin [28] and many others at the LMB. Since then, the screen has had a clear impact in other laboratories notably during investigations of proteins required for the outer kinetocore assembly [29] and the function of an RNA-silencing complex [30]. Although the MORPHEUS screens were initially intended for soluble proteins, they can also be useful for transmembrane proteins [31], essentially because of their PEG-based precipitants and low salt concentrations (a similar observation was made earlier about the Pi-PEG screen).

## ANGSTROM optimization screen

To bypass the formation of ice crystals during flash-cooling with liquid nitrogen or cold nitrogen gas, crystals can be pre-equilibrated by soaking in a solution containing a cryo-protectant, in many cases glycerol. Because the crystal structure of a protein is typically held together by a restricted number of weak intermolecular interactions, it can easily be damaged or lost because of different cooling rates and expansion coefficients between the crystal and the surrounding liquid [32]. With a multitude of possible interactions with water and proteins via different spatial arrangements of hydroxyl groups, polyols are ideal components to alter parameters of protein crystallization and flash cooling of crystals. For example, polyols have a capacity for water adsorption [33] and hence will alter crystallization mechanisms and the kinetics of equilibration during vapor-diffusion experiments. They can also enhance the stability of proteins [34]. In these respects polyols lend themselves as crystallization additives.

**Figure 4:**
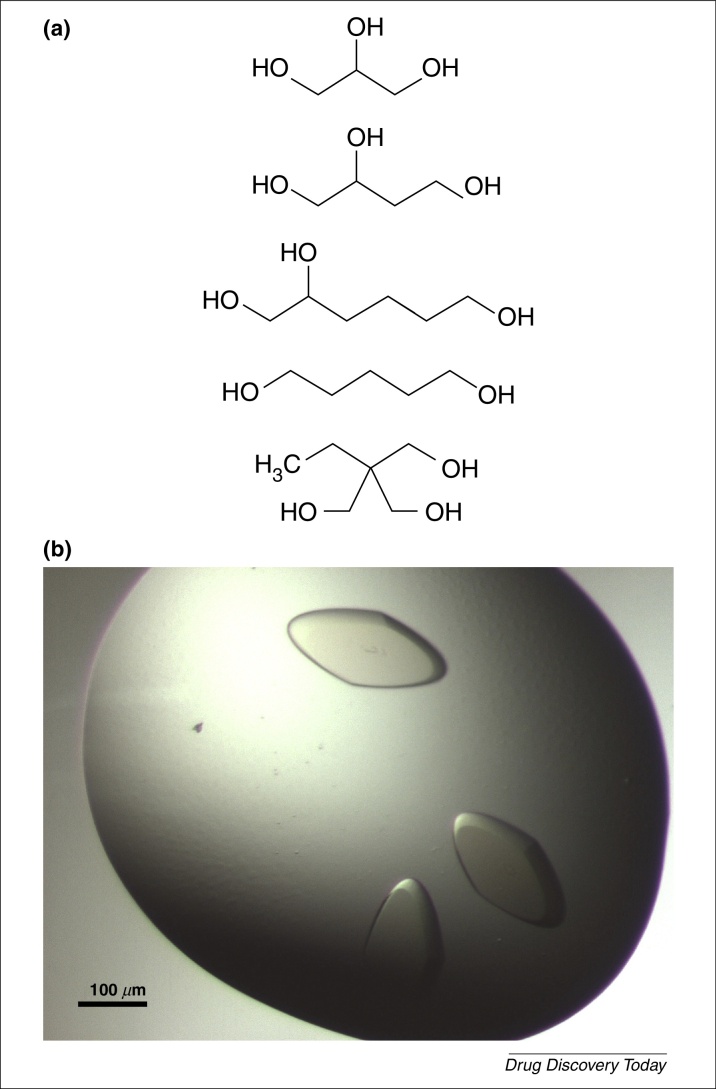
**(a)** Glycol derivatives. From top to bottom: 1,2,3-propanetriol (glycerol), 1,2,4-butanetriol, 1,2,6-hexanetriol, 1,5-pentanediol and 1,1,1-tris(hydroxymethyl)propane. In addition to being well-suited as crystallization reagents, these five polyols are cryo-protectants when used at concentrations as low as 20–25% (w/v). **(b)** Light photographs of crystals of endosomal sorting complex required for transport (ESCRT)-I. Ten percent of the ANGSTROM screen was added to the reservoirs of a crystallization plate pre-filled with 96 repeats of the initial condition. Several hits were observed including the one shown here with glucose as additive (final conc. 3%, w/v). Unpublished results obtained during the early stage of the ANGSTROM screen development. Work of Nicolas Soler (LMB).

After testing over 100 commercially available polyols, we found about a third act as cryo-protectants, although many cryo-protecting polyols were not as potent as glycerol (*i.e.* cryo-protectant concentrations of 20–25%, w/v). The ANGSTROM screen was later formulated with 31 cryoprotecting polyols at different concentrations (Table S1, see supplementary material online) – essentially derivatives of glycols (Fig. 4a), carbohydrates and PEGs. Fig. 4b shows an example of a successful optimization experiment with a sample of endosomal sorting complex required for transport (ESCRT)-I.

## Discussion

An underlying problem when developing a new screen is the very large number of variables that makes comparison and validation difficult or even impossible to achieve. Ultimately, there are never enough samples or conditions when trying to investigate the causal relationships that can govern crystallization of proteins. I would argue that MORPHEUS and Pi sampling are innovative tools because they enable the formulation of unique screens that are highly efficient. However, it is important to acknowledge earlier work that was a source of inspiration while designing these new tools, notably the developments of Alexander McPherson and Bob Cudney 7, 8, 35, 36, 37. Further developments to integrate more heavy atoms [25] and cryo-protectants [26] into crystallization protocols will be especially useful. For example, chelates used to solubilize heavy atoms could further facilitate novel structure solution [38]. Other formulations to find solutions for transmembrane protein crystallization [39] and electron cryomicroscopy of two-dimensional crystals [40] are being developed. Ideally, a new chemistry that opens the way for innovative approaches needs to be introduced 41, 42, 43, 44.

Producing protein variants is of course a major strategy to solve a structure nowadays, notably with very advanced recombinant DNA technologies [45], surface engineering [46] and limited proteolysis [47]. Furthermore, the often limited amounts of sample and cost-effectiveness should not be ignored. In this context, the optimization of a reduced set of conditions is important [48]. We however regularly observe the benefits of employing a wide range of screens with proteins that were reluctant to crystallize (or formed crystals that could not be exploited). Subsequently, I would argue that progress in macromolecular crystallography depends on further miniaturization of crystallization experiments to reduce costs and enable the use of very large screens as another main strategy 49, 50, 51.

## Concluding remarks

Theoretical and pragmatic aspects were taken into account to develop innovative protein crystallization screens. Because the search space associated with diffraction-quality protein crystals is almost infinite, the process investigated was considered as stochastic. We hence formulated screens *de novo* with reagents highly represented in crystal structures (although the LMB sparse matrix was more safely formulated with a selection of pre-existing conditions known to be successful).

Formulations were tested for protein stabilization, crystallization and crystal screening with protein crystallization standards (that crystallize readily) and challenging samples available at the time at the LMB. We regularly obtained exclusive and useful hits in the new screens, that means the corresponding developments had a very positive impact on our structure-determination process. To increase our yield of diffraction-quality crystals further, a footprint approach to formulation [52] with innovative features is being developed (the “Delta screen”).

## Conflicts of interest

We hereby state a conflicting commercial interest because MRC Technology commercializes the screens presented here.
